# Kaempferide Prevents Titanium Particle Induced Osteolysis by Suppressing JNK Activation during Osteoclast Formation

**DOI:** 10.1038/s41598-017-16853-w

**Published:** 2017-11-30

**Authors:** Zixian Jiao, Weifeng Xu, Jisi Zheng, Pei Shen, An Qin, Shanyong Zhang, Chi Yang

**Affiliations:** 1 Department of oral and maxillofacial surgery, Shanghai Ninth People’s Hospital, Shanghai Jiaotong University School of Medicine, Shanghai, 200011 China; 2 Department of Orthopedics, Shanghai Key Laboratory of Orthopedic Implant, Shanghai Ninth People’s Hospital, Shanghai Jiaotong University School of Medicine, Shanghai, 200011 China

## Abstract

Kaempferide (KF) is an O-methylated flavonol, a natural plant extract, which is often found in Kaempferia galanga. It has a variety of effects including anti-carcinogenic, anti-inflammatory, anti-oxidant, anti-bacterial and anti-viral properties. In this study, we aimed to investigate whether KF effectively inhibits titanium particle induced calvarial bone loss via down regulation of the JNK signaling pathway. In the mice with titanium particle induced calvarial osteolysis, the Low dose of KF mildly reduced the resorption pits while in the high dose group, fewer scattered pits were observed on the surface of calvarium. Histological examination showed fewer osteoclasts formation in the KF group. In mouse bone marrow macrophages (BMMs) and RAW264.7 cells, KF significantly inhibited the osteoclast formation and bone resorption at 12.5 μM. However, KF does not affect the mature osteoclast F-actin ring formation. But when being co-treated with KF and anisomycin, BMMs differentiated into mature osteoclasts. At the molecular levels, the JNK phosphorylation was inhibited and the osteoclastogenesis-related specific gene expression including V-ATPase d2, TRAP, calcitonin receptor (CTR), c-Fos and NFATc1 was markedly suppressed. In conclusion, these results indicated that KF is a promising agent in the treatment of osteoclast-related diseases.

## Introduction

End-stage temporomandibular joint (TMJ) diseases such as osteoarthritis, severe inflammatory condylar resorption, idiopathic condylar resorption and TMJ ankylosis usually result in loss of the posterior vertical height of the mandible and therefore need TMJ reconstruction. At present, the commonly used treatment modalities for such diseases include autogenous bone grafts^1^ such as costochondral graft, sternoclavicular graft, and coronoid graft or total joint replacement (TJR) with artificial prosthesis. TJR of the TMJ is an effective treatment for an intractable pain and impaired TMJ function^2^. However, those patients are often relatively young (30 to 35 years of age) and need long term use of prosthesis^3^. Wear particles such as titanium particles generated from prosthesis can cause long term complication such as aseptic peri-prosthesis loosening. Indeed, the loosening and instability of the condylar component and the fixation screws of the TJR are one of the most widely reported complications associated with TMJ prosthetic replacement^4^. Therefore, the prevention of aseptic loosening of TMJ prosthesis takes on added importance.

Theoretically, the long term use of the prosthesis will cause the release of small wear particles between the bone and the implant interface. The released titanium particles can thus recruit and activate macrophages, resulting in the release of different inflammatory mediators such as IL-1β, IL-6, IL-17 and TNF-alpha^5,6^, which in turn can enhance the expression of RANKL from the surrounding osteocytes and stromal cells^7^. The increased RANKL levels can subsequently activate osteoclast formation and bone resorption, leading to periprostheic bone loss and therefore causing prosthetic loosening and instability^8^.

Accordingly, there are two ways for improving the clinical outcome of the total joint prosthesis: 1) Synthesis of more biocompatible prosthesis materials that can reduce the release of wear particles. 2) Searching for compounds that can inhibit macrophage and/or osteoclast activation. During the screening of such compounds that can inhibit osteoclast formation and function, we identified a natural compound derived from the roots of kaempferia galanga, kaempferide (KF) and found KF is capable of suppressing osteoclast function. Previous studies showed that KF has a series of biological activities including antioxidant^9,10^ and antibacterial^11^ properties. However, to our knowledge, there are no reports discussing the role of KF on bone metabolism and titanium particles induced osteolysis. Furthermore, the possible use of KF in preventing osteolysis *in vivo* remains unclear. Therefore, this study aimed to investigate whether KF has an inhibitory effect on titanium particle induced osteolysis *in vivo* and to unveil its mode of action *in vitro*.

## Results

### KF inhibited osteoclast differentiation *in vitro*

**Figure 1:**
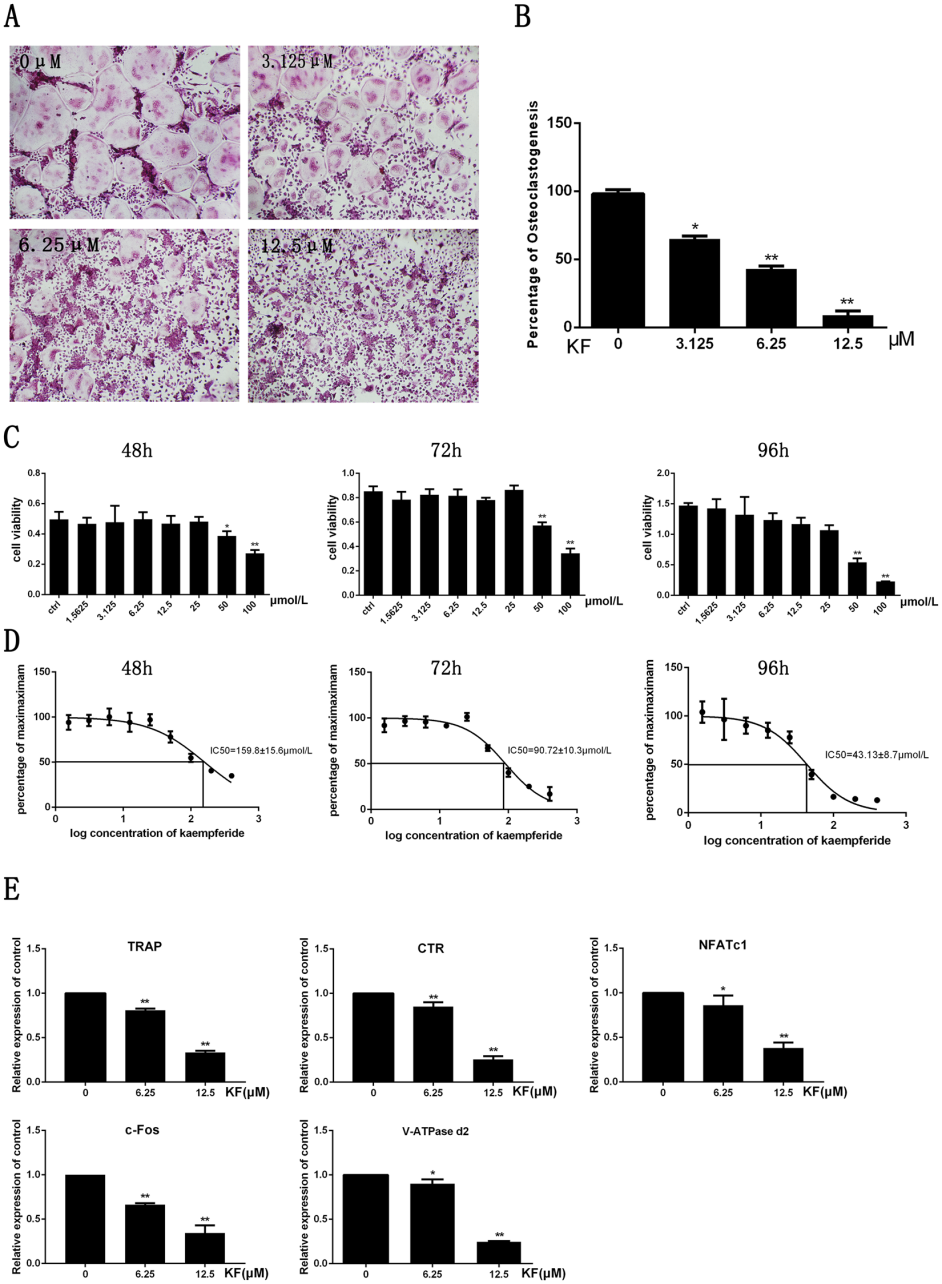
KF inhibits RANKL-induced osteoclast formation *in vitro*. (**A**) BMMs were treated with various concentrations of KF followed by 30 ng/ml M-CSF and 50 ng/ml RANKL, after incubation for 7 days, cells were fixed with 4% paraformaldehyde and subjected to TRAP staining. (**B**) Number of TRAP-positive multinucleated osteoclasts. (**C**) KF shows no cytotoxicity at low concentrations. Viability of KF treated BMM cells after being incubated for 48 h, 72 h and 96 h. (**D**) The half-maximal inhibitory concentration (IC50) of kaempferide was 159.8 ± 15.6 μM, 90.72 ± 10.3 μM and 43.13 ± 8.7 μM, respectively. (**E**) KF suppresses RANKL-induced gene expression. BMM cells were cultured with M-CSF (30 ng/ml), RANKL (50 ng/ml) and KF (6.25 μM and 12.5 μM) for 5 days. RANKL-inducible gene expression was analyzed by real time PCR. RNA levels were normalized relative to the expression of Beta-actin (*p < 0.05, **p < 0.01).

First, we investigated the effect of KF on osteoclast differentiation *in vitro*. As shown in Fig. 1A, a large number of TRAP-positive multinucleated osteoclasts formed in the control group, while the presence of KF inhibited osteoclast formation in a dose dependent manner. Treatment of osteoclasts with KF at 3.125 μM mildly inhibited osteoclast formation, with approximately 30% reduction in the number of osteoclast formation. Compared with the control group, the addition of KF at 12.5 μM significantly suppressed osteoclast formation, with almost no round osteoclast formed in this group. There are only about 10.16 ± 4.22 osteoclasts formed in the 12.5 μM group. (Fig. 1B). Collectively, KF inhibited osteoclast differentiation in a dose dependent manner.

### KF had no cytotoxicity at low concentrations

In order to exclude the possibily of KF on osteoclast differentiation is not due to the cytotoxicity of KF on osteoclasts, the effect of KF on cell viability was evaluated. As shown in Fig. 1C, KF exhibited cytotoxic effect on the osteoclast precursor cells at 50 μM or higher concentrations after the incubation for 48 h, 72 h and 96 h respectively. The IC_50_ of KF was 159.8 ± 15.6 μM, 90.72 ± 10.3 μM, and 43.13 ± 8.7 μM at 48 h, 72 h and 96 h respectively (Fig. 1D). No cytotoxic effects were observed at 25 μM or lower concentrations. Thus, KF has cytotoxic effect on osteoclast precursor cells at concentrations ≥50 μM. Since we observed KF at 12.5 μM had dramatic inhibitory effect on osteoclast differentiation. Therefore, the inhibitory effect of KF on osteoclastogenesis is not due to its cytotoxic effect.

### KF suppressed RANKL-induced gene expression *in vitro*

The suppression of osteoclast differentiation is further evaluated by examination the osteoclast specific gene expression profile. Osteoclasts treated with different doses of KF were harvested for RNA extraction and real-time PCR. As shown in Fig. 1E, the expression of osteoclast specific TRAP gene was expressed in the control group. However, its expression was significantly inhibited after being treated with KF. Approximately 50% reduction was noticed. Similarly, another osteoclast specific marker CTR demonstrated similar expression trend, suggesting a reduced osteoclast number after KF treatment. In addition to these mature osteoclast markers, the expression of key transcription factors NFATc1 and c-fos were also inhibited after KF treatment, which result in the attenuated expression of their downstream gene expression such as V-ATPase d2 (Fig. 1E). Taken together, our realtime PCR results confirmed the inhibitory effect of KF on osteoclast differentiation *in vitro*.

### KF inhibited the function of osteoclasts: bone resorption assay

**Figure 2:**
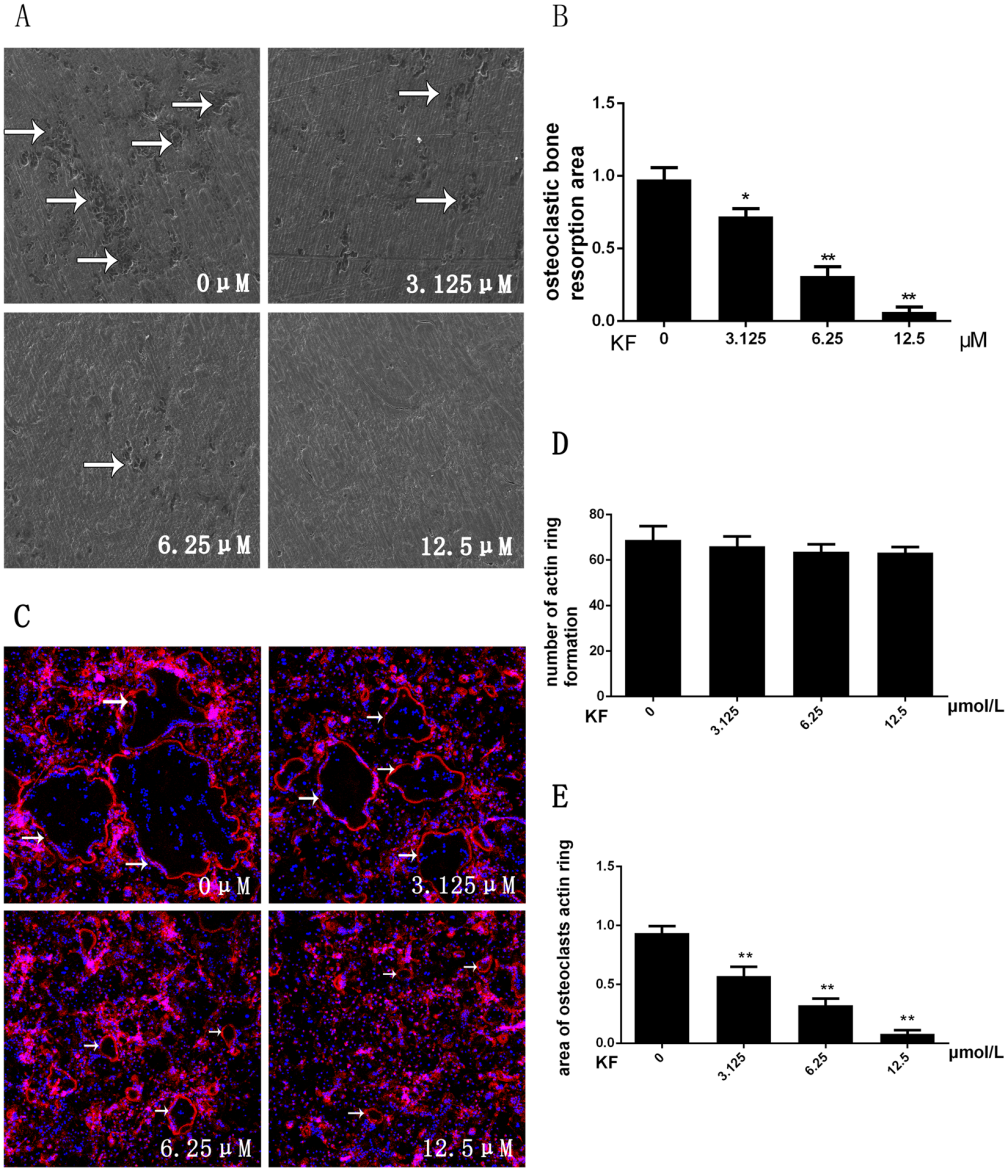
(**A**) BMM cells were seeded onto bovine bone slices, the cells were treated with M-CSF (30 ng/ml), RANKL (50 ng/ml) and KF (0, 3.125, 6.25 or 12.5 μM) until the formation of osteoclasts. Bone resorption pits were examined by S.E.M. (**B**) Bone-resorption pits area were measured using image J software and are presented graphically. (**C**) KF suppresses RANKL-induced acting ring formation. BMM cells were treated with RANKL (50 ng/ml) and KF (0, 3.125, 6.25 or 12.5 μM), after osteoclasts were formed, the cells were fixed and stained for laser scanning confocal microscope assay. (**D**) The number of formed actin ring was not affected with the increasing concentration of KF. (**E**) KF suppresses the area of osteoclasts actin ring. (*p < 0.05, **p < 0.01).

Since osteoclast differentiation was inhibited, we assumed that KF can subsequently inhibit the osteoclast function. Thus, the osteoclast precursors were seeded on the surface of bone slices with the absence or presence of KF at different concentrations. Scanning electron microscope (SEM) showed that large areas of bone resorption pits were observed in the control group. The percentage of the resorption area in the control group was significantly higher than in the KF group. A dose dependent suppressive effect of KF on osteoclast bone resorption was noticed, while nearly no resorption pits were observed at 12.5 μM (Fig. 2A). Further analysis of the bone resorption area using Image J software also confirmed the inhibitory effect of KF on bone resorption *in vitro*. About 60% and 90% reduction of bone resorption area were observed in the groups treated with KF at 6.25 μm and 12.5 μM respectively (Fig. 2B). Collectively, these data indicated that KF inhibited osteoclast bone resorption *in vitro*.

### KF does not affect the RANKL-induced F-actin ring formation

Prior to osteoclast induced bone resorption, the differentiated osteoclasts need to reconstruct their cyto-skeletal structure, known as F-actin ring formation. Thus, we then investigated whether KF can affect F-actin ring formation *in vitro*. As shown in Fig. 2C, under the circumstances of M-CSF (30 ng/ml) and RANKL (50 ng/ml) that induced the mature osteoclast formation, a well-structured F-actin ring was observed in the control group. In agreement with the suppressed osteoclast differentiation as mentioned above, the number of F-actin ring was indeed reduced after being treated with different doses of KF. However, we can still observe the well-preserved ring-like structures in the drug treated groups at 3.125 μM and 6.25 μM. When KF concentration was increased to 12.5 μM, almost no mature large osteoclasts were detectable. However, small ring-like structures still existed. Thus, our results suggested KF does not affect the mature osteoclast F-actin ring formation.

### KF depressed the osteoclastogenesis via down regulating the JNK and ERK signaling pathway

**Figure 3:**
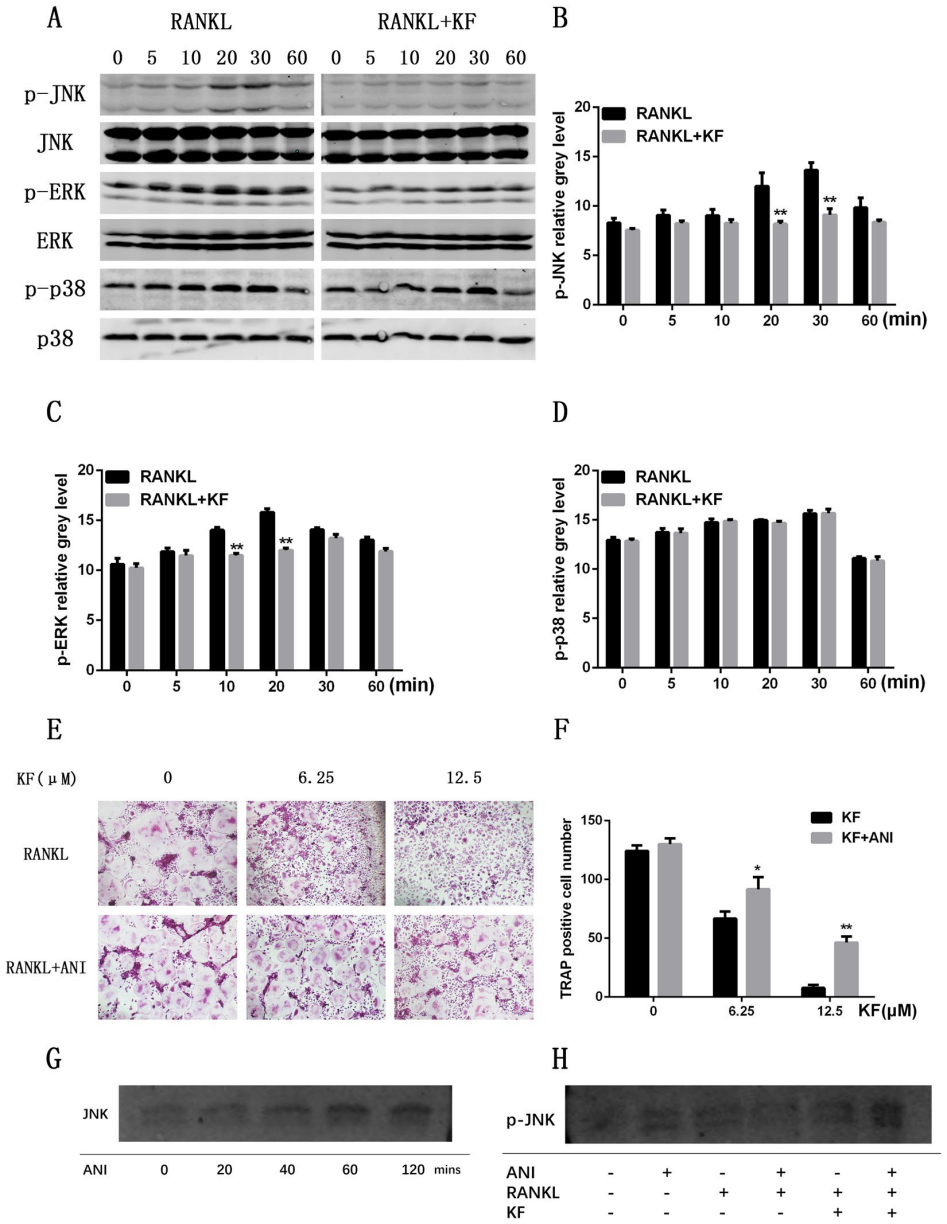
RAW264.7 cells were pretreated with vehicle or KF for 4 h followed by RANKL stimulation (50 ng/ml) for the indicated times. Then cells were lysed in lysis buffer, and lysates were analyzed by Western blotting with the indicated antibodies. (**A**) KF treatment suppressed the phosphorylation of JNK and ERK signaling pathways, while the phosphorylation of p38 was not inhibited. (**B**) Statistical analysis of p-JNK grey level showed significant difference between the KF treatment group and the control group at 20 and 30 minutes. (**C**) KF also attenuated the ERK phosphorylation at 10 and 20 minutes. (**D**) KF treatment does not affect p38 signaling pathway. (**E**) The inhibitory effect of KF on osteoclastogenesis was rescued by anisomycin. When treated with RANKL and KF, osteoclast formation was significantly inhibited, this inhibitory effect was rescued by anisomycin. (**F**) In each KF group with same concentration, the number of osteoclast increased significantly after treatment of anisomycin. (**G**) The JNK expression increased gradually from 0 to 120 mins. (**H**) When treated with RANKL+KF+Anisomycin, the p-JNK expression was observed compared to the RANKL+KF group.

All the aforementioned results indicate that KF can suppress the osteoclast differentiation and thus inhibiting bone resorption *in vitro*. However, the underlying molecular mechanisms on how KF affects the osteoclastogenesis require further investigations. MAPK signaling pathways are known to play pivotal roles in the osteoclast differentiation. Thus, we further checked the activation of these signaling pathways with the presence or absence of KF. As shown in Fig. 3A, the RANKL-induced JNK phosphorylation was observed in the control group, peaking at 20 min and 30 min. However, the activation of JNK phosphorylation was significantly suppressed after KF treatment. The expression of p-JNK in the KF group was significantly lower than that in the control group as reflected by the statistical analysis (Fig. 3B). We also examined the effect of KF on other MAPK signaling pathways including p38 and ERK phosphorylation. As shown in Fig. 3A, the phosphorylation of ERK was also observed in the control group. Similarly, KF partially attenuated the ERK phosphorylation as compared to the control group (Fig. 3C). Different from JNK and ERK, the phosphorylation of p38 was not affected after KF treatment (Fig. 3D). However, the NF-kB pathway, which is another downstream signaling pathway, was not affected (data not shown). The above results showed that KF inhibited the phosphorylation and degradation of JNK and ERK, without affecting the p38 signaling pathway.

### The inhibitory role of KF on osteoclastogenesis was rescued by anisomycin

In order to further confirm that KF suppressed the osteoclast formation by affecting the JNK activation, we further employed a rescue assay to validate this. As shown in Fig. 3E, the osteoclast formation was significantly restrained when the cells were treated with KF only. In contrast, the addition of the JNK agonist, anisomycin, rescued osteoclastogenesis, where mature osteoclasts were formed (Fig. 3E). Statistical analysis showed that when the BMMs treated with KF and anisomycin, both at 6.25 μM and 12.5 μM concentrations, the number of osteoclasts was significantly higher than that of the KF treated group (Fig. 3F). Western bolt showed that the JNK phosphorylation was rescued, as shown in Fig. 3H, when treated with RANKL+KF+Anisomycin, the p-JNK expression was observed compared to the RANKL+KF group, which means the JNK phosphorylation was rescued. At the same time, the administration of anisomycin didn’t affect JNK protein synthesis (Fig. 3H), the JNK expression increased gradually from 0 to 120 mins (Fig. 3G). Together, these data implicated KF suppressed osteoclast formation via inhibiting the JNK signaling pathway.

### Interaction between KF and the JNK/ERK protein

**Figure 4:**
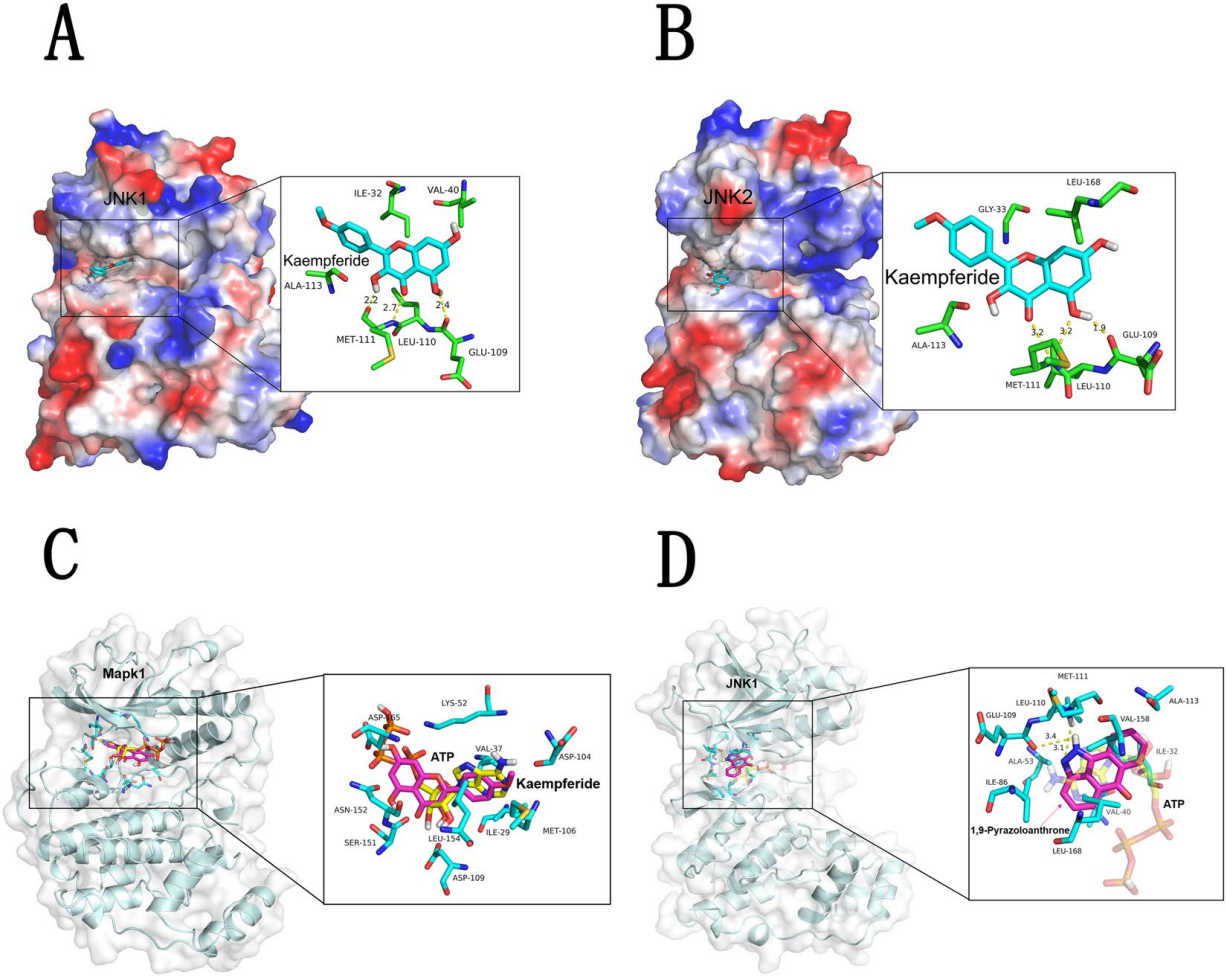
(**A**–**C**) Molecular docking model produced by PyMOL visualization software showed the probable combining site of KF with JNK1/JNK2 and ERK. (**D**) Interactions of 1,9-Pyrazoloanthrone and JNK.

As the phosphorylation of JNK is an important molecular step in the process of osteoclast differentiation, we next examined whether KF can bind to JNK protein based on a computational calculation. As shown in the molecular docking, KF could form stable connections with JNK1 (Fig. 4A) and JNK2 (Fig. 4B) ATP binding sites. Specifically, KF interacted with MET-111, LEU-110 and GLU-109 of JNK1 and JNK2. However, we failed to find the potential binding sites of KF onto ERK protein (Fig. 4C). Together with the western blotting, the molecular docking indicated that KF suppressed the phosphorylation of JNK. In order to testify that the other JNK inhibitor might express the similar effects as that of KF, we also examined the possible interaction of 1,9-Pyrazoloanthrone, which is a specific JNK inhibitor, with JNK. As shown in the Fig. 4D, 1,9-Pyrazoloanthrone could form stable connections with JNK ATP binding sites at LEU-110 and MET-111, which expressed the similar effect as that of KF.

### KF suppressed Ti-particle induced osteolysis *in vivo*

**Figure 5:**
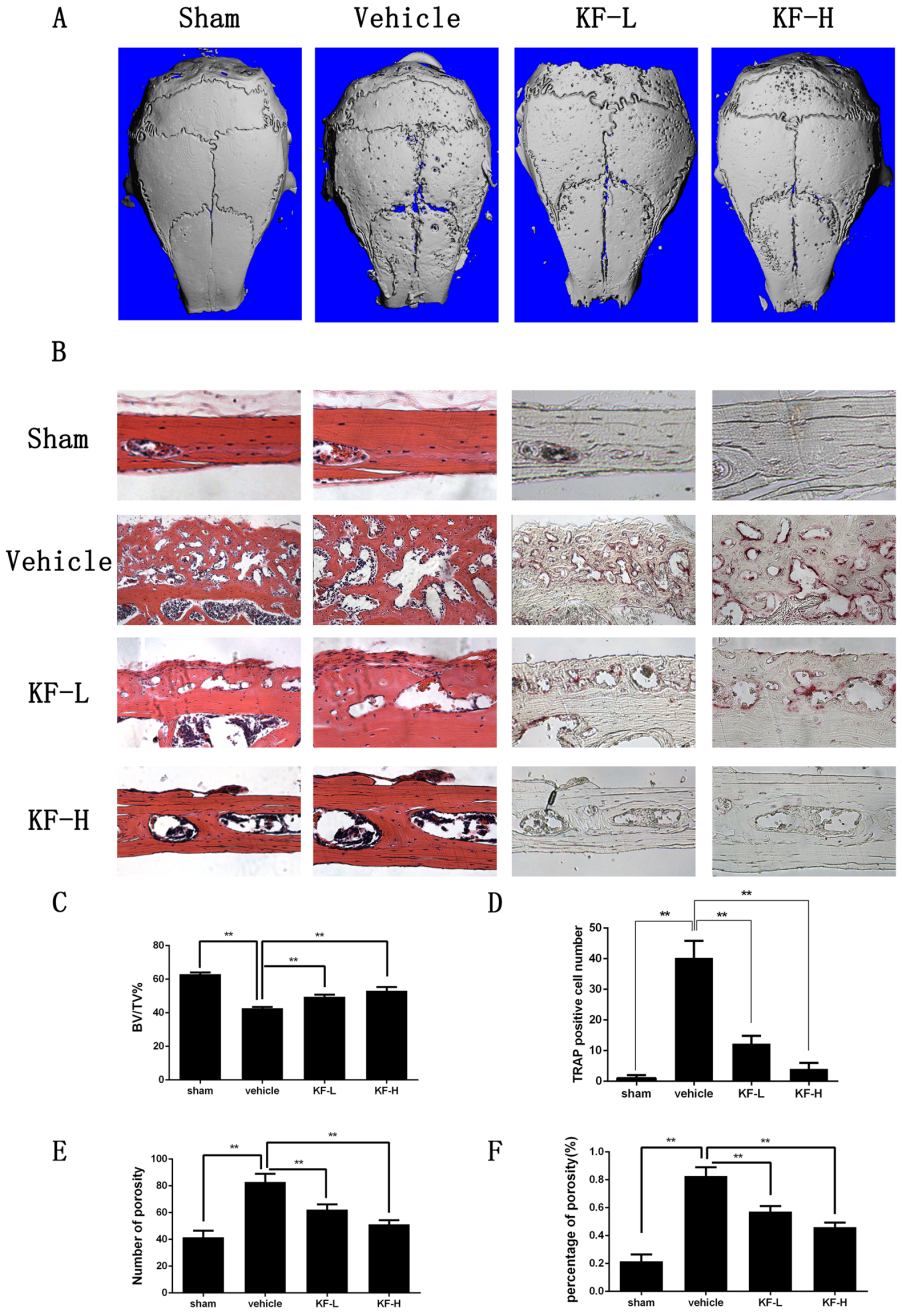
Kaempferide (KF) suppresses Ti-particle induced osteolysis. (**A**) Micro-CT scanning showed that the loss of bone volume on KF injection group was significantly less than that of vehicle group. (**B**) Both HE and TRAP staining indicated an inhibitory effect of KF on osteolysis, the vehicle group showed an obvious inflammatory reaction and notably osteolysis, while the KF injection groups reveled reduced inflammation and osteolysis. (**C**) Bone volume against tissue volume (BV/TV), number of TRAP positive osteoclasts, number of porosity and the total porosity of each sample was measured.

Our *in vitro* experiments suggested KF is effective in preventing the formation and function of osteoclasts. To validate the potential therapeutic effect of KF on preventing titanium particles induced bone loss, we then administered KF in titanium induced osteolysis model *in vivo*. As shown in Fig. 5A, reconstruction of micro-CT scanning showed an extensive bone loss after titanium stimulation in the vehicle group, with numerous large and deep resorption pits on the calvarial surface. In contrast, the calvarial surface was relatively smooth in the sham group. Interestingly, the administration of KF attenuated particle induced osteolysis. Low dose of KF (4 mg/kg/day) mildly reduced the resorption pits, with fewer and smaller pits on the calvarial surface. More obvious attenuation of titanium induced osteolysis was observed in the high dose group (8 mg/kg/day), with fewer scattered pits observed on the surface, especially along the suture line. Statistical analysis of bone volume/total volume (BV/TV), number of porosity and the percentage of total porosity in the region of interest (ROI) also confirmed the Micro-CT scanning results. On the sham group, BV/TV was significantly higher than that of the vehicle group, while the value of BV/TV was increased with the presence of KF (Fig. 5C). On the vehicle group, both the number of porosity and the percentage of porosity were greatly higher than the other 3 groups. With the increasing concentration of KF, the value of the KF-L and the KF-H group were significantly decreased.

Furthermore, the histological examination confirmed the protective effect of KF on Ti-particle induced osteolysis. Extensive bone destruction was observed in the vehicle group, with a lot of TRAP positive osteoclasts on the surface of the dissolved bone tissue (Fig. 5B). However, the number of mature osteoclasts in KF-L and KF-H group was significantly reduced (Fig. 5D). In addition, no fatalities were recorded and all mice remained with normal activity during the whole experiment. Together, these data indicated KF is capable of preventing titanium induced osteolysis *in vivo*.

## Discussion

For many end-stage diseases of the TMJ such as severe osteoarthritis, ankylosis, idiopathic condylar resorption and tumors involving the mandibular condyle, TJR with an artificial prosthesis is the most effective way to treat these diseases. However, aseptic loosening of the prosthesis is one of the most important causes of failure for hip and knee joint prostheses^12^, and this complication most likely will be the same for TMJ total joint prostheses. Because TMJ patients are often relatively young (30 to 35 years of age), a total TMJ prosthesis must have a very long lifetime^3^. The prevention of aseptic loosening of the TMJ prosthesis gains much more attention recently. Generally, the titanium particle induced loosening is due to the periprosthesis osteolysis induced by an activation of a large number of osteoclasts. Therefore, osteoclasts inhibitors are thought to be potential drugs for the titanium particle induced osteolysis.

Flavonoids are natural phenolic compounds present in fruits and vegetables with antioxidant^13^, anti-carcinogenic^14^ and other biological functions^15,16^ including osteogenic and anti-osteoclastogenic effects. Eerduna *et al*.^17^ found that flavonoids reduce the myocardial infarction size after an artery ligation in rats, through regulating the antioxidative enzymes activity and the endothelial nitric oxide synthase activity. Flavonoids also have neuroprotective actions, having the potential as multi-targeted therapeutic tools for protecting the brain^18^. Recently, being a kind of Flavonoids, kaempferol, an analogue of KF, has been shown to have an inhibitory role on the bone loss in mice long bones by preventing the osteoclast formation^19^. However, whether KF has the same role on osteoclast formation and bone resorption both *in vivo* and *in vitro* has not been discussed before.

In our study, KF was shown to have an inhibitory effect on osteoclast formation and function in a dose dependent manner. At the concentration of 12.5 μM, osteoclastogenesis was inhibited obviously and almost no resorption pits were observed on bone slices. The inhibited bone resorption is most likely due to suppressed osteoclast formation, as supported by the fact that KF suppressed TRAP positive ostgeoclast formation and gene expression in a dose dependent manner. These findings can further reflect the *in vivo* findings that the administration of KF can prevent osteoclastic bone loss by reducing TRAP positive osteoclast number in our titanium particle induced osteolysis model.

Furthermore, the molecular mechanisms underlying this inhibitory effect of KF were elucidated. Western blotting revealed that KF inhibited the RANKL-induced JNK and ERK signaling pathways without affecting the p38 signaling pathway. During the osteoclast metabolism process, JNK plays a very important role^20^. Johnson *et al*. found that c-Jun–deficient mice are embryonic lethal^21^. Ikeda also found that the body size of the transgenic mice in which the domain-negative c-Jun lacking the transcriptional activation domain was much smaller than that of the wild type mice. Also the transgenic mice showed an increased radio density of the long bones, jaw bones, and vertebrae compared with the control mice. An activated JNK subsequently leads to the activation of the transcription factor c-Jun^22^. C-Jun together with c-Fos, an essential transcription factor for osteoclast formation, can form the activator protein-1 (AP-1) complexes. AP-1 can bind to the NFATc1 promotor and regulate its expression. NFATc1 is a master regulator in osteoclastogenesis process^23^, and the coupling of c-Jun signaling with NFAT family is crucial for the transcriptional events during osteoclastogenesis^24^. NFATc1 can regulate the expression of several genes associated with osteoclast differentiation and function. *In vitro* promotor analyses identified the nuclear factor of activated T-cells (NFAT)/AP-1 sites in the osteoclast-specific Acp5 (TRAP) and Calcr (CTR) promotors. In addition, the transcriptional induction of NFATc1 is considered a major function of c-Fos in osteoclast differentiation^25^. In this study, KF inhibited JNK phosphorylation as demonstrated by WB results. This is further evidenced by our molecular docking assay that KF can interact with the ATP binding site of MET-111, LEU-110 and GLU-109 of JNK1 and JNK2. Again, the addition of JNK agonist anisomycin reversed the inhibitory effect of KF on osteoclast formation. All the results point to the possible mode of action that KF suppressed JNK activation and lead to impaired osteoclast formation.

In summary, our study demonstrated that KF is able to prevent titanium particle induced osteolysis *in vivo* and inhibit osteoclastogenesis *in vitro*, and the inhibitory effect can be played at a lower concentrations. KF could be considered as a potential agent for the prevention of particle induced osteolysis in future. Surface modification or local injection of this natural compound is of potential in the treatment of peri-prosthesis loosening. Also, KF is also used in Chinese cooking and traditional Chinese medicine, so it can be intake from the daily diet. Admittedly, there are some limits of this study. The effect of KF on osteoblast and osteocyte biology needs further investigation. For future translation of this finding into clinic, surface coating of this natural compound onto prosthesis need further validation. Another classical animal model for studying osteoclasts is the collagen-induced arthritis in mice, which will be performed in the further study of elucidating inflammatory lesions of TMJ such as TMJ osteoarthritis. Generally, we demonstrated natural compound KF is of value in Ti-particle induced osteolysis.

## Materials and Methods

### Media and reagents

KF was obtained from Meilun (Dalian, Liaoning, China) and then it was dissolved in Dimethylsulfoxide (DMSO) with a concentration of 100 mM stock solution. Alpha-MEM, fetal bovine serum (FBS), and penicillin were purchased from Gibco BRL (Gaithersburg, MD, USA). Soluble mouse recombinant M-CSF and RANKL were purchased from R&D Systems (USA). Tartrate-resistant acid phosphatase (TRAP) staining solution was from Sigma–Aldrich. The Cell Counting Kit-8 (CCK-8) was obtained from Dojindo Molecular Technology (Japan). Primary antibodies targeting GADPH, phospho-ERK, ERK, phospho-JNK, JNK, phospho-p38, p38, NF-kB and IkB-a were purchased from Cell Signaling Technology (CST, Danvers, MA, USA). The Prime Script RT reagent Kit and SYBR® Premix Ex Taq™ II were obtained from TaKaRa Biotechnology (Otsu, Shiga, Japan).

### Cell culture

BMMs were prepared according to the method of Qin^26–28^. Briefly, cells extracted from the femur and tibiae of a 6-week-old C57/BL6 mouse were incubated in complete cell culture media with 30 ng/ml M-CSF in a T-75 cm^2^ flask for proliferation. RAW264.7 cells were cultured in α-MEM containing 10% FBS, 2 mM L-glutamine, 100 U/ml penicillin/streptomycin. The cell cultures were maintained at 37 °C in a humid environment with 5% CO_2_^29^.

### Cell viability assay

The anti-proliferative effect of KF on BMM cells was assessed with cell counting kit-8 (CCK-8, Dojindo Laboratories, Kumamoto, Japan) following the manufacturer’s instructions^30^. BMM cells were seeded at a density of 1 × 10^4^ cells/well supplied with complete α-MEM, M-CSF (30 ng/ml) and increasing concentrations of KF (0, 1.5625, 3.125, 6.25, 12.5, 25, 50, 100, 200, 400 μM). Following the treatment with KF for 48 h, 72 h and 96 h respectively, 10 μl CCK-8 solution was added to each well; the cells were then incubated for 4 h and absorbance was measured at 450 nm and 630 nm using a microplate reader. The half-maximal inhibitory concentration (IC_50_) value was calculated by GraphPad Prism program version 6.01. The effect of KF on cell viability was expressed as percent cell viability with the vehicle-treated control cells set at 100%^31,32^.

### *In vitro* osteoclastogenesis assay

BMM cells were seeded onto a 96-well plate at a density of 8 × 10^3^ cells/well in triplicate and supplied with complete α-MEM medium. The cells were treated with M-CSF (30 ng/ml), RANKL (50 ng/ml) and various concentrations of KF (0, 3.125, 6.25 or 12.5 μM). The culture medium was replaced every 2 days until the formation of osteoclasts was noticed at day 7. Then the cells were fixed with 4% paraformaldehyde for 20 min and stained with TRAP using the Diagnostic Acid Phosphatase kit. TRAP positive cells with more than 3 nuclei were counted as osteoclasts.

### Quantitative PCR analysis

For real-time PCR, 5 × 10^4^ BMMs were seeded in a 6-well plate and cultured in a complete medium containing α-MEM, 10% FBS, 100 U/mL penicillin, M-CSF (30 ng/mL), and RANKL (50 ng/mL). Cells were treated with KF (6.25 μM or 12.5 μM) for 7 days. After formation of osteoclasts, the total RNA was extracted using the Qiagen Rneasy Mini kit (Qiagen, Victoria, Australia). A single-stranded cDNA was synthesized from 2 μg of the total RNA using the iScript cDNA Synthesis Kit (Bio-Rad, Hercules, CA). Subsequently, a real-time PCR was performed on an ABI 7500 Sequencing Detection System using the SYBR® Premix Ex Taq™ II. In short, 10 μl of SYBR® Premix Ex Taq™ II, 7.2 μl ddH2O, 2 μl cDNA and 0.4 μl of each primer were mixed to make up a total volume of 20 μl for each PCR. Cycling condition was 95 °C 5 s and 60 °C 34 s for 40 cycles with specific primers for V-ATPase d2, TRAP, CTR, c-Fos and NFATc1. Beta-actin was included as housekeeping gene. The comparative 2^−ΔΔCT^ method was used to calculate the relative expression levels of each gene as previously described^33^. The following primer sets were used as previously described^34–37^: mouse Beta-actin: forward, 5′-TCTGCTGGAAGGTGGACAGT-3′ and reverse, 5′-CCTCTATGCCAACACAGTGC-3′; mouse NFATc1: forward, 5′-CCGTTGCTTCCAGAAAATAACA-3′ and reverse, 5′-TGTGGGATGTGAACTCGGAA-3′; mouse CTR: forward, 5′-TGCAGACAACTCTTGGTTGG-3′ and reverse, 5′-TCGGTTTCTTCTCCTCTGGA-3′; mouse c-Fos: forward, 5′-CCAGTCAAGAGCATCAGCAA-3′ and reverse, 5′-AAGTAGTGCAGCCCGGAGTA-3′; mouse V-ATPase d2: forward,5′-AAGCCTTTGTTTGACGCTGT-3′ and reverse 5′-TTCGATGCCTCTGTGAGATG-3′; TRAP: forward 5′-CTGGAGTGCACGATGCCAGCGACA-3′ and reverse 5′-TCCGTGCTCGGCGATGGACCAGA-3′.

### Bone resorption assay

For the bone resorption assay, BMM cells were seeded onto bovine bone slices in a 96-well plate with complete α-MEM at a density of 2.4 × 10^4^ cells/cm^2^. The cells were treated with M-CSF (30 ng/ml), RANKL (50 ng/ml) and KF (0, 3.125, 6.25 or 12.5 μM) for 7 days. Then osteoclasts were removed by mechanical agitation and sonication. The resorption pits were visualized under a scanning electron microscope (SEM, FEI Quanta 250). The percentage of resorbed bone surface area was quantified using the Image J software (National Institutes of Health). Briefly, the bone resorption pits were quantitatively measured using “Measuring Area” tool: first, surround an area with a perimeter. This can be done with an area selection tool, then select “Analyze → Measure” transfers the area measurement to a data window.

### Actin ring-formation assay

The BMM cells (8 × 10^3^ cells/well) were seeded onto dentine slices in a 48-well plate at a density of 2.4 × 10^4^ cells/cm^2^. Cells were treated consistently with the bone resorption assay. Briefly, after osteoclasts were formed, the cells were washed three times with ice-cold PBS, fixed in 4% formalin, and then permeabilized by the incubation in 0.1% Triton-PBS for 15 min. The cells were then blocked with 1% bovine serum albumin–PBS, and incubated with FITC-labeled phalloidin for 30 min. The cells were extensively washed with PBS, and the nuclei were stained with 4′, 6-diamidino-2-phenylindole (DAPI). Cover slips were mounted on a microscope slide with an embedding medium, and F-actin rings formation was visualized using a fluorescence microscope (Leica)^38,39^. For each dentine slice, five random fields were selected and the number of complete actin rings were counted, then the total number of actin ring formation was recorded. Each dentine slice was counted for 3 times and the average number was calculated as the number of actin ring formation. The area of actin ring formation was quantitatively measured using Image J software (National Institutes of Health) “Measuring Area” tool: first, surround an area with a perimeter. This can be done with an area selection tool, then select “Analyze → Measure” transfers the area measurement to a data window.

### Western blot analysis

RAW264.7 cells were pretreated with a serum-free α-MEM with/without KF for 4 h, and then stimulated with RANKL at 0, 5, 10, 20, 30, and 60 min. After being washed twice in 1× PBS, the cells were lysed in ice-cold lysis buffer containing 50 mM Tris–HCl, 150 mM NaCl, 5 mM EDTA, 1% Triton X-100, 1 mM sodium fluoride, 1 mM sodium vanadate, 1% deoxycholate, and protease inhibitor cocktail. Then the lysate was centrifuged at 12,000 rounds/minute for 12 min and the protein in the supernatant was collected. Protein concentrations were measured through the BCA assay. Equal amounts of the protein lysates were separated using a 10% SDS–PAGE and then transferred to polyvinylidene difluoride membranes (Millipore, Bedford, MA, USA). These membranes were then blocked with 5% (g/ml) skim milk solution for 1 h and probed with the primary antibodies (GAPDH, 1:1000; phospho-ERK1/2, 1:1000; ERK1/2, 1:1000; phospho-JNK, 1:1000; JNK,1:1000; phospho-p38,1:1000; p38, 1:1000; NF-kB, 1:1000, IkB-a, 1:1000) overnight at 4 °C. The membranes were incubated with the appropriate secondary antibodies conjugated with IRDye 800CW (molecular weight, 1166 Da; LI-COR, Lincoln, NE, USA), meanwhile the antibody reactivity was detected by the exposure in an Odyssey infrared imaging system (LI-COR).

### Osteoclastogenisis rescue assay

BMM cells were seeded onto a 96-well plate at a density of 8 × 10^3^ cells/well in triplicate and supplied with complete α-MEM medium. After adhering to the well, M-CSF (30 ng/ml), RANKL (50 ng/ml) and various concentrations of KF (0, 6.25 or 12.5 μM) were added to each well. In addition, a rescue group was treated with KF and a potent activator of JNK, anisomycin. After the mature osteoclasts formed at day 7, the cells were fixed and stained with TRAP, and the number of TRAP-positive cells was then counted. Then western blot analysis was performed to further verify the rescue effect of anisomycin. Briefly, BMM cells were treated with RANKL, RANKL+KF, RANKL+KF+anisomycin, the p-JNK expression was evaluated. At the same time, in order to exclude the effect of anisomycin on protein synthesis, JNK synthesis was evaluated after administration of anisomycin at 0, 20, 40, 60, 120 mins.

### Molecular Modeling

Homology models of mouse JNK1/JNK2 kinase domain and Mapk1 (Erk) kinase domain were built with MODELLER 9.12^40^, using the structure of the human JNK1/JNK2 (PDB code: 2H96, 3E7O) and Mapk1 (Erk) as a template. The models were further evaluated for the stereochemical quality using PROCHECK^41^. The three-dimensional coordinates for KF was generated and optimized with Marvin Sketch and molconvert packages from ChemAxon (http://www.chemaxon.com/). Ligands were docked to the ATP binding pocket of JNK and Mapk1 (Erk) by AutoDock and AutoDock Vina^42^. Ligand conformation search was based on Lamarckian genetic algorithm. All the default parameters were used during the docking after protein preparation. Predicted binding mode figures were prepared with PyMOL visualization software (http://www.pymol.org).

### Animal model of Titanium particle-induced calvarial osteolysis

The Animal Care and Experiment Committee of Shanghai Jiao Tong University School of Medicine approved all experimental procedures, and the study was carried out according to the guidelines for the Ethical Conduct in the Care and Use of Nonhuman Animals in Research by the American Psychological Association.

Briefly, 32 8-week-old C57/BL6 mice were randomly assigned into 4 groups: sham operation group (sham), Ti-particle with phosphate-buffered saline (PBS, vehicle), Ti-particle with low concentration of KF (KF-L, 4 mg/kg/day) and high concentration of KF (KF-H, 8 mg/kg/day). Pretreatment of Ti particle was performed according to the method of Liu as early reported^37,43^. Then the cranial periosteum was separated from the calvarium by sharp dissection and 30 mg of Ti-particle were embedded under the periosteum at the middle suture of the calvarium. Within the following 14 days, mice in the KF-L and KF-H groups were injected with different concentrations of KF (4 or 8 mg/kg/day respectively) every other day. The other 2 groups received PBS injection every other day. After 2 weeks of injection, the mice were sacrificed, and the calvaria were excised and fixed in 4% paraformaldehyde for micro-CT analysis.

### Micro-CT scanning

The micro-CT scanning was carried out using a high-resolution micro-CT (SCANCO 100, SCANCO, Brüttisellen, Switzerland). The resolution of the scanning was 10 μm, and the X-ray energy was set at 70 kvp, 200 μA. After 3D reconstruction, the calvaria samples were decalcified in 10% EDTA for further studies.

### Histological and histomorphometric analysis

After being decalcified in 10% EDTA for 3 weeks, the calvaria were embedded in paraffin, and histological staining including hematoxylin and eosin staining (H&E) and TRAP-staining was performed. The slices were then examined and photographed under a high-quality microscope and the TRAP-positive multinucleated cell was considered as osteoclast.

### Statistical analysis

The data were expressed as the means ± SD. Results were analyzed with Student’s t-test using the SPSS 13.0 software (SPSS Inc., USA). *p* < 0.05 indicated a significant difference between the groups.

### Equipment and settings

Figures 1 A and 3E were obtained using DP Manager version 1,1,1,71. Figure 2A showed the bone resorption pits were visualized under a scanning electron microscope (SEM, FEI Quanta 250). The percentage of resorbed bone surface area was quantified using the Image J software (National Institutes of Health). Figure 2B showed the actin ring formation was visualized using a fluorescence microscope (Leica) and the area of actin ring formation was quantified using Image J software (National Institutes of Health). Figure 3A,G and H representing the western blot gels were obtained by Odyssey infrared imaging system (LI-COR) application software version 3.0.16. In Fig. 4, the predicted binding mode figures were prepared with PyMOL visualization software (http://www.pymol.org). Figure 5 was the analysis of animal model, the figures of micro-CT scanning were obtained using a high-resolution micro-CT (SCANCO 100, SCANCO, Brüttisellen, Switzerland). The histological figures were obtained using LEICA DM4000 B microscope. Figures of statistical analysis including Figs 1B,C,D,E, 2B,D,E, 3B,C,F, and 5C,D,E,F were obtained using GraphPad Prism program version 6.01.
